# Valuing Citizen Access to Digital Health Services: Applied Value-Based Outcomes in the Canadian Context and Tools for Modernizing Health Systems

**DOI:** 10.2196/12277

**Published:** 2019-06-06

**Authors:** Christina Hackett, Kelsey Brennan, Heather Smith Fowler, Chad Leaver

**Affiliations:** 1 Social Research and Demonstration Corporation Ottawa, ON Canada; 2 Canada Health Infoway Toronto, ON Canada

**Keywords:** personal health records, patient portals, electronic health records, patient engagement, health care costs, cost of illness, economic evaluation

## Abstract

**Background:**

In publicly funded health systems, digital health technologies are strategies that aim to improve the quality and safety of health care service delivery and enhance patient experiences and outcomes. In Canada, governments and health organizations have invested in digital health technologies such as personal health records (PHRs) and other electronic service functionalities and innovation across provincial and territorial health systems.

**Objective:**

Patients’ access to their own information via secure, Web-based PHRs and integrated virtual care services are promising mechanisms for supporting patient engagement in health care. We draw on current evidence to develop an economic model that estimates the demonstrated and potential value of these digital health initiatives.

**Methods:**

We first synthesized results from a variety of Canadian and international studies on the outcomes for patients and service providers associated with PHRs across a continuum of services, ranging from viewing information (eg, laboratory results) on the Web to electronic prescription renewal to email or video conferencing with care teams and providers. We then developed a quantitative model of estimated value, grounded in these demonstrated benefits and citizen use (2016-2017). In addition to estimating the costs saved from patient and system perspectives, we used a novel application of a compensating differential approach to assess the value (independent of costs) to society of improved health and well-being resulting from PHR use.

**Results:**

Patients’ access to a range of digital PHR functions generated value for Canadians and health systems by increasing health system productivity, and improving access to and quality of health care provided. As opportunities increased to interact and engage with health care providers via PHR functions, the marginal value generated by utilization of PHR functionalities also increased. Web-based prescription renewal generated the largest share of the total current value from the patient perspective. From the health systems perspective, Canadians’ ability to view their information on the Web was the largest value share. If PHRs were to be implemented with more integrated virtual care services, the value generated from populations with chronic illnesses such as severe and persistent mental illness and diabetes could amount to between Can $800 million and Can $1 billion per year across Canadian health systems.

**Conclusions:**

PHRs with higher interactivity could yield substantial potential value from wider implementation in Canada and increased adoption rates in certain target groups—namely, high-frequency health system users and their caregivers. Further research is needed to tie PHR use to health outcomes across PHR functions, care settings, and patient populations.

## Introduction

### Health Systems and Digital Health Technologies

Digital health technology is an umbrella term encompassing a variety of innovations such as mobile health, health information technology, wearable devices, telehealth and telemedicine, and personalized medicine. Digital health technologies are envisioned as a step forward in empowering patients to participate in their health care decisions and, thereby, *democratize* health care. In publicly funded health systems, digital health technologies are strategies aimed at improving the quality and safety of health care service delivery as well as enhancing patient experiences and outcomes [1-3]. For example, the United Kingdom and Australia have launched large-scale personal health records (PHRs) initiatives—the NHS.UK and My Health Record, respectively—to connect patients with their electronic health and medical records [4-6].

In Canada, federal and provincial governments and health organizations have invested in digital health technologies and innovation across provincial and territorial health systems, including PHRs with a variety of electronic service (e-service) functionalities [7]. Canada Health Infoway is a federally funded, nonprofit organization whose mandate is to “realize the vision of healthier Canadians through innovative digital health solutions.” Through Infoway, Canada has invested in the development, implementation, and evaluation of various digital health solutions, including PHRs.

### Personal Health Records and Health System Improvement

A PHR is defined as a digital space, or Web/mobile, application-based, health or medical record that holds all or a portion of clinical information about an individual (eg, laboratory results or prescribed medications) [8-11]. Multiple terms exist to refer to platforms through which patients can access their health care information, including PHR, patient portal, or consumer portal (to maintain consistency, we use PHR throughout). PHRs can be either stand-alone and driven by information entered by individuals and stored on their personal devices or tethered to a health care organization or system. Information shared via PHRs can come from multiple sources and is managed, shared, and controlled by the individual patient; therefore, PHRs should conform to nationally recognized interoperability standards [12].

PHRs in the scope of analyses presented in this paper:

1. are integrated with point-of-care clinical health information systems, allowing individuals to view their health record information from electronic medical records, hospital (or hospital consortium) information systems, laboratory testing and results information systems, electronic health records (EHRs), or health information exchanges;
2. are tethered to a health care organization or system, and are linked to information provided within the EHR attached to that system (hospital, insurance plan, or other health care organization); and
3. contain connected care functionalities/services such as communication and consultation features with point-of-care clinics, providers, or care organizations.

Stand-alone PHRs were considered out of scope, including goal- or outcome-defined clinician-patient coaching models that allow patients to input information and PHRs that do not link to clinical-source information systems.

**Figure 1:**
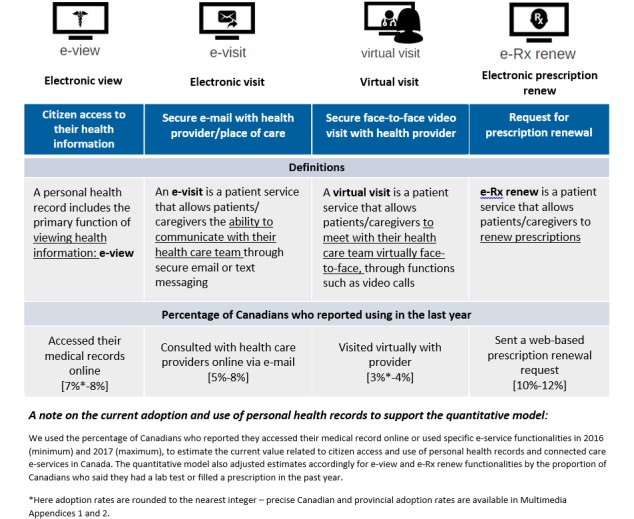
Definition and utilization of electronic service functionalities in Canada (2016-2017).

Patients access their PHRs through a secure personal identification authorization process. PHRs can provide a range of e-service functionalities, categorized in-depth by the Center for Information Technology Leadership as comprising 4 overarching types of informational interaction—collection, sharing, self-management, and exchange [13]. *How* PHRs facilitate these interactions can vary; functions include the ability to (1) view health care information (electronic view [e-View]), (2) exchange secure emails and messages with health care providers (electronic visit [e-Visit]), (3) renew prescription medication on the Web (electronic prescription renew [e-Rx renew]), and (4) visit virtually via videoconference with their health care providers (virtual visit). These functionalities are illustrated in Figure 1, alongside Canadian adoption and use rates from 2016 and 2017. As such, PHRs have the potential to influence value-based outcomes in terms of avoided direct and indirect costs but also as a mechanism to improve longer-term trends in health literacy, patient satisfaction [14], access and user-centered design [15-17], and clinical outcomes.

The terms p activation, engagement, empowerment, and patient-centered care, although used and measured distinctly, are often used interchangeably and broadly refer to mechanisms by which patients and caregivers interact with their care trajectory and the degree to which they feel confident and satisfied in doing so [18]. Efforts to enhance patient activation and patient engagement in health and health care, as well as improve patient experience, are increasingly at the forefront of health policy and strategy in both high-resource and low- to middle-income health systems worldwide [19-21].

A growing body of literature highlights how PHRs can increase patient activation by providing seamless access points through which patients access their health care information and communicate with their providers or care teams [11,22,23]. Patients’ interaction with their health care information has been shown to positively influence patient engagement, health behaviors, and associated health outcomes in a variety of settings [24-26]. However, this body of work has focused largely on patient activation in health care and health outcomes and less on the *process* by which activation occurs through engagement with digital health solutions [27-29]. For the remainder of the paper, we use the term *patient activation* to refer to the desired outcome of patients’ interactions with their PHR and *patient engagement* as the mechanism through which PHRs facilitate patient activation.

PHRs have been shown to influence patient engagement by reducing barriers to access to information via seamless Web-based platforms [18]. In certain high-volume health management organizations in the United States (eg, Kaiser Permanente and Veterans Affairs), where tethered PHRs have been implemented and value-based outcomes evaluated, PHR functionalities involving high levels of patient interaction with their regular health care providers (via e-Visits and virtual visits) have been shown to improve health outcomes, while reducing per capita costs of care [21,41]. Despite these benefits, it is important to note that a digital divide exists between those who can and do access these technologies and those for whom there are barriers to using technology to engage with their health care provider. Differences in access are influenced by multiple sociodemographic factors. However, there is little consensus about determinants of access and uptake across intersections of individual-level characteristics: often there are differences in use by socioeconomic status, education, and age, those in lower-income quintiles having lower levels of education and those of older age tend to access digital health technologies less [30-32]. Physical access to internet and to technological devices, in addition to technology-related skills and literacy, all contribute to perpetuating or bridging the digital divide in high-resource contexts [33,34]. A recent electronic health equity framework notes the complex interplay of macro (socio-techno-economic-political context), patient (social position and intersection between various demographics), intermediary (material circumstances and social capital), and digital technology implementation factors that influence access to health technologies [35].

### Evaluation of Value-Based Outcomes and Digital Health Technology/Personal Health Records

Although there are a growing number of economic evaluations of digital health solutions, there are conceptual, methodological, and practical challenges to assessing the economic benefit of implementing and adopting PHRs, both in terms of increased health system efficiency and improved quality of and access to medical care [10,11]. These challenges include the lack of a clearly defined perspective resulting from multiple investors and sources of funding, the lack of identified options for comparison, and having comparable costs and outcomes across different PHR interventions/sites [11,36]. In addition, economic analyses and evaluations often take place in single health care settings with a focus on a specific patient population, and are not easily interpretable at a systems level. Conversely, analyses focusing solely on the benefits of PHRs and associated cost-savings or financial outcomes for a broader population are often based on relatively few sources of evidence, which may or may not reflect the health system contexts in which the benefit estimates are applied.

Using an established approach developed by DeLone and McLean [37] for evaluating information technology initiatives, Canada Health Infoway (Infoway) has developed a comprehensive benefits evaluation framework for sites implementing PHRs, inclusive of net benefits or value-based outcomes [38]. This framework facilitates the evaluation of implementation factors as well as aligning outcomes with PHR use.

Our objectives in this study were as follows:

1. To synthesize outcomes generated by benefits evaluations conducted at multiple sites implementing PHRs in Canada, across different types of care settings, and serving different patient populations.
2. To estimate the relative economic benefit of implementing PHRs in those different care settings and patient populations compared with business as usual (either before or in the absence of PHR implementation) from 3 perspectives—health system (payer), patient and caregiver, and the economic benefit to society resulting from improved population health (societal perspective).

We used contextually specific data as well as cost and outcome data from the peer-reviewed literature to demonstrate the current and potential added value generated by PHR adoption and implementation at a national level across various health care settings, patient populations, and 4 specific PHR functionalities. In addition to estimating value based on resources saved by patients and caregivers and clinician and clinic productivity gains, we used a novel application of various approaches to empirically link PHR use and improved health, health behaviors, and increased life satisfaction.

## Methods

### Overview

We developed 3 quantitative models to estimate the economic benefit of patients and health care organizations having access to PHRs, relative to what occurred or would occur in the absence of being able to access PHRs (ie, business as usual). Typically, economic analyses of interventions or technologies in health care estimate the comparative effectiveness, utility, or broader opportunity cost between 2 or more alternative interventions [11,39]. Given the variability of implementation costs, relative provincial investment in digital health initiatives, and amount of financial support allocated to each PHR implementation site in Canada, we opted to present a cost-outcome description that synthesizes the aggregate population-level economic benefits of PHR adoption and implementation in Canada. Specifically, we applied a 1-sided economic analysis that estimates the economic benefits of the relative health care quality, access, and productivity gains from PHR implementation versus nonimplementation. Each of the 3 models reflects a different perspective from which we conducted our analyses:

1. Patient and caregiver perspective
2. Health systems perspective
3. Societal perspective/improved health

### Data and Model Inputs

We drew on the benefits evaluation studies of recent PHR initiatives conducted by Canadian health care organizations (n=12) to derive estimates of any added economic benefits. Data from these evaluations represented PHR sites in 5 provinces (British Columbia, Saskatchewan, Ontario, Quebec, and Nova Scotia). Each study employed a system and use survey that examined the use of PHR functions as well as PHR user experiences and also explored quantitative outcomes of use. In addition, the sites used administrative records and user reports to assess the odds of user versus nonuser requests for health care information, average number of visits avoided (for both groups), and resulting organizational efficiency gains (eg, full-time equivalent staffing saved). Further information regarding the studies included in our 3 quantitative models can be found in Multimedia Appendices 1 and 2.

To expand our models to include relevant benefits found in the peer-reviewed literature, we conducted a systematic search of the following electronic bibliographic databases: EconLit, Health Systems Evidence, PsycINFO, and MEDLINE. Our key search terms included personal health record OR patient portal AND (economic benefit OR benefit). The following limits were applied to all searches: (1) keyword search to abstract and title, (2) English or French, and (3) 2010 to 2017. A team of 2 researchers conducted the searches and subsequent stages of review and extraction.

#### Selection of Model Inputs

For inclusion in our study, the peer-reviewed and gray literature had to (1) provide estimates of the economic benefit of PHR use from the patient, system, or societal perspectives, (2) provide clinical benefits of PHR use, and (3) take place in a high-resource health system context. The excluded studies were those without a counterfactual scenario (ie, PHR use compared with no health system use), those that included PHRs with functionalities out of scope, or intervention studies without a quantified clinical outcome clearly attributable to PHR use. After applying the selection criteria, a total of 81 studies were selected for full-text review, and 21 of these were used as the primary input sources to the quantitative models developed.

From studies selected for inclusion (including the benefits evaluations conducted in Canada), 2 researchers reviewed each document and extracted data on clinical and economic benefits of PHR use. A third member of the research team (CL) validated each extracted benefit and model input. The outcomes reported by respondents in these evaluations generated a range of values of effectiveness, using a series of classification factors in the Infoway Benefits Evaluation framework. As seen in Table 1, these classification fields included PHR functionality, care setting, benefit domain, and benefit recipient or patient population targeted by the intervention and care setting.

**Table 1:** Classification factors for benefit estimation.

Field of classification		Definition
**PHR^a^** **functionality**		**The method by which the PHR engages patients/caregivers.**
	Electronic view	Primary function of PHR is the viewing of health information.
	Electronic visit	A patient e-service that allows patients and their caregivers the ability to communicate with their health care team through secure email or short message service text messaging.
	Virtual visit	A patient e-service that allows patients and their caregivers the ability to meet with their health care provider via a face-to-face virtual encounter through functions such as video calls.
	Electronic prescription renew	A patient e-service that allows patients and their caregivers to renew prescriptions.
**Care setting**		**The medical care setting in which the benefit of PHR was found to accrue.**
	Primary care	Day-to-day health care delivered by a health care provider (eg, general practitioner’s office).
	Specialist care—mental health	Health care provided for issues related to mental health, including community-based and inpatient care.
	Specialist care—chronic conditions	Health care provided for issues related to other chronic conditions such as diabetes.
	Hospital-based care	Inpatient and outpatient care provided in hospital/hospital-affiliated settings.
	Pediatric care	Health care provided to children.
**Benefit domain**		**Areas of value.**
	Quality	An increase in health quality as a result of PHR use, such as increased healthy behaviors, improved health outcomes, or increased life satisfaction.
	Productivity	An increase in productivity as a result of PHR use, such as saved time or resources.
	Access	An increase in access to health care as a result of PHR use.
**Benefit recipient**		**The recipient of the benefit.**
	Patient/caregiver	The benefit accrued directly to the patient or caregiver.
	Health system	The benefit accrued to the health system (eg, the primary care provider or the hospital).
	Health outcomes	The benefit involved an improvement in population health.
**Resource saved**		**The way in which patients/caregivers, the health system, and health outcomes benefitted from PHR use.**
	Avoided visits to health care providers	The primary way by which patients/caregivers benefitted from PHR use, including saved time and cost related to travel, saved time arranging caregiving and caregiving costs, and avoided time off work.
	Increased productivity among health systems	The primary way by which health systems benefitted from PHR use, including avoided visits and reduced calls from patients/caregivers, avoided emergency department visits, and avoided preventable adverse drug events.
	Improved healthy behaviors	A way in which health outcomes benefitted from PHR use, such as better medication adherence.
	Increased life satisfaction	A way in which health outcomes benefitted from PHR use, using a validated life satisfaction scale.
	Improved health	A way in which health outcomes benefitted from PHR use, through changes in patient activation.

^a^PHR: personal health record.

**Figure 2:**
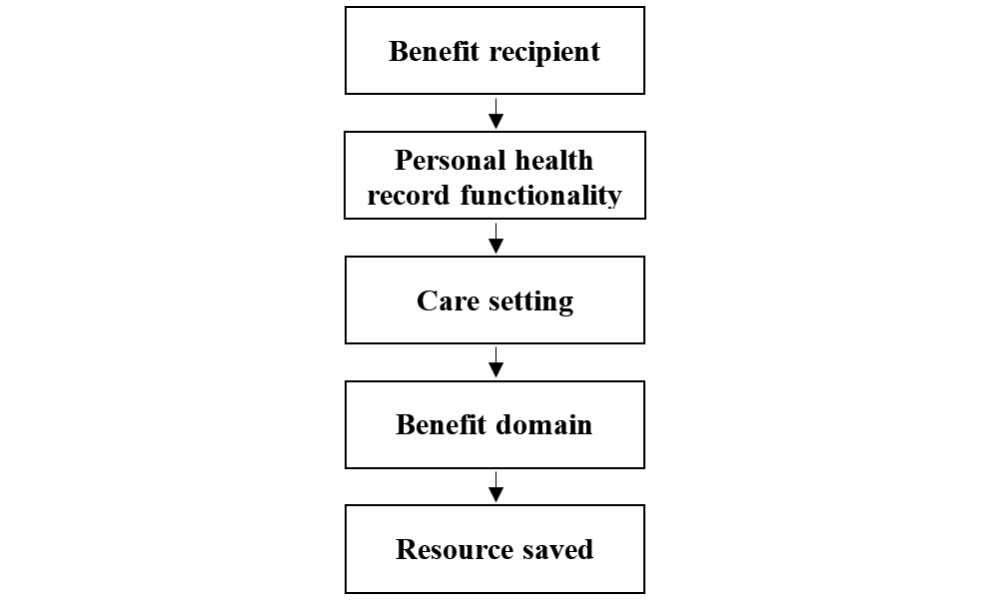
Hierarchy of benefit classification.

Once we classified areas of value to benefit domains, as outlined above, we created a hierarchy of specific value-based outcomes, as outlined in Figure 2, such that benefit recipients were the primary classification factor, followed by the associated PHR functionality, care setting, benefit domain, and empirical value of resource saved. Estimates were adjusted to the specific yearly (2016 and 2017) utilization rates for each PHR functionality, the Canadian population aged over 18 years reporting access or use of indicated health services, and affected clinical populations for health outcomes estimates, both nationally and by care setting.

As it is unknown if and to what extent certain PHR functionalities might increase health care costs, we defined and included value-based outcomes in terms of the change in units of resources used (by patients and health systems) relative to a counterfactual scenario. The extracted outcomes data represented the added value of PHR use reported compared with an alternative scenario or counterfactual. For example, 1 health system outcome was *avoided medical errors* achieved through patient use of an electronic prescription renewal when compared with traditional prescription renewal processes. The value to the health system in this regard results from reduced preventable adverse events (primarily drug-related events) and a resulting reduction in unnecessary health system utilization. To avoid double-counting benefits or discounting costs related to PHR implementation in terms of patient and health system resources, we included only benefits that were clearly separate from resource costs. For example, because e-Visit functionalities in primary care settings involve physician time, we did not count avoided in-person visits to primary care physicians as an economic benefit to the health system but did for patients who saved time and financial costs.

Multimedia Appendices 3, 4, and 5 provide an overview of the included Infoway studies, the final sample of respondents from which the reported outcomes are derived, the counterfactual/comparison group or alternative scenario used for comparison, and the type of indicators used for the patient, health system, and societal perspectives, respectively. In addition to the outcomes extracted from Canadian PHR initiatives, we drew on various sources to identify relevant estimates for classification factors from publicly available data. See Multimedia Appendices 1 and 2 for an overview of the data sources used to estimate economic benefits Canada-wide from patient/caregiver and health system cost perspectives, respectively.

### Model Estimation

We defined the economic benefit or value *V* to each perspective as the reported costs to patients and health systems in the absence of PHRs, less the cost savings *S* reported as a result of PHR adoption or implementation within a health care setting. We offset the cost savings reported by subtracting an estimate of the deadweight, or cost savings that may have occurred naturally, in the absence of PHR adoption.

V_Patient_ = C_PCurrent_ – (S_T_ + S_LF_ + S_C_ – D)

Costs to patients and caregivers were expenses related to attending an in-person appointment with their health care provider. Resources saved by patients included travel costs and travel time, caregiving costs and time spent arranging care, and reduced time away from paid employment. Direct average costs were obtained from primary data collected from patients’ and caregivers’ self-reporting from Canadian PHR implementations and used as model inputs. In terms of indirect costs, the amount of time saved was obtained from patients’ and caregivers’ self-reporting and valued using median Canadian income for time away from paid employment. For time costs that did not relate to paid employment, time was valued at 25% and 50% of median income. Multimedia Appendix 1 contains details related to the sources used to value patient/caregiver costs, and Multimedia Appendix 6 provides a narrative summary of how we calculated estimates from this perspective.

V_Health system_ = C_HSCurrent_ – (S_U(AV,H0)_ + S_PG_ + S_PS_ – D)

From the health system perspective, current costs are a function of the quantity of health care services provided within the relevant health care setting and to the relevant patient population and the cost per service. Resources saved for health systems include health care provider time (reduction in clinician time to complete tasks/patient consultation), increased productivity, and savings resulting from improved patient safety (eg, reduced preventable adverse drug events). For example, patients and caregivers having access to prescription information on the Web can increase patient and caregiver awareness of their current and previous medications, resulting in decreased prescription error on the supply side (pharmacists and health care providers) and increased medication adherence by patients. See Multimedia Appendices 2 and 7 for details regarding valuation sources and an overview of methods used to estimate the value of citizens’ use of PHR functionalities to health systems in Canada.

V_Population_ = C_HSCurrent_ – (V_Satisfaction_ + S_Health_ + S_Health behavior_ – D)

Where there are tangible costs avoided to the health system attributable to PHR use (eg, unnecessary in-person visits avoided and provider time on operational tasks mitigated by Web-based communication or automation), estimates of benefit to the health care systems in Canada are more straightforward than for less tangible benefits such as improved health, health care behaviors, and health status. To provide estimates of improvements to behaviors and health status, we converted positive health outcomes reported in the amassed evidence base into specific econometric indicators of resources saved, according to associated PHR functionalities.

In addition to estimating the value of improved health behavior and health status of PHR users compared with nonusers, we created a direct empirical link between improvement in life satisfaction reported by adults with severe and persistent mental illness as a result of using PHR functionalities and a household income equivalent. We applied a compensating differential approach developed by Helliwell and Huang (2010) [40] that takes the difference in life satisfaction before/after for PHR users versus nonusers and then divides this by the coefficient for the marginal effect of life satisfaction on household income for Canadians (beta=0.14). For a detailed overview of the approaches used to estimate the value of improved health behaviors and health status, see Multimedia Appendix 8.

### Range of Values

In each of the 3 models, we estimated a range of value for benefits currently realized as well as the potential value for these initial benefit areas with advanced utilization rates. Ranges in current utilization rates of available PHR functionalities were measured in 2016 and 2017 from nationally representative surveys designed by Canada Health Infoway and administered by an independent research organization [7,8]. To model potential value-based outcomes, we applied projected utilization rates of 25%, 35%, and 50%. The magnitude of ranges illustrated in our final estimates represent variations in the reported outcomes realized, in the way in which personal time can be valued as a proportion of income, and in reported uses of the health care system.

### Assumptions

The assumptions underlying our calculations were as follows:

1. The benefits realized by the study sample from source evidence reflect benefits that could be realized by populations with similar case mixes or diagnoses.
2. Benefits realized by health care organizations in terms of increased productivity are transferable to other similar contexts across Canada.
3. The proportion of the population represented by each province in Canada can be used to expand province-level benefits to national-level benefits. Conversely, national-level benefits can be disaggregated into those at the provincial-level.
4. Outcomes related to PHR effectiveness remain constant over time.

All the estimates generated by valuing provider time (ie, time saved by health care providers and organizations) were created on the basis of a range of values from the least expensive unit of time (eg, an administrative staff) to the most expensive unit of time (eg, a physician).

## Results

### Overview

Table 2 summarizes the outcomes identified in our review by PHR functionality and outlines areas in which PHRs have led to identified benefits and in which care settings those benefits were realized. Current Canadian PHR adoption rates range from 3% to 4% for virtual visit technology to 10% to 12% for e-Rx renew functionalities. The benefits realized included avoided visits and time costs saved across different care settings, such as community-based clinics, primary care clinics, and hospitals.

**Table 2:** Summary of evidence of Canadians’ current use of personal health records and electronic services.

Personal health record functionality	Electronic view	Electronic visit	Virtual visit	Electronic prescription renew
Adoption rate	7%-8% of Canadians can and have accessed their health care information on the Web.	5%-8% of Canadians can and have communicated with their health care provider securely on the Web.	3%-4% of Canadians can and have visited virtually with their health care provider securely on the Web.	10%-12% of Canadians can and have renewed their prescription medication on the Web.
Care setting	Primary care, hospital care, and community-based mental health services were care settings where Canadians and health systems benefited from accessing their health care information on the Web.	Community-based mental health services were the care setting where Canadians and health systems benefited from communicating with their health care provider securely on the Web.	Primary care was the care setting where Canadians and health systems currently benefited from visiting virtually with their health care provider on the Web. Primary care networks for people with chronic conditions demonstrated potential benefits to Canadians and health systems.	Community-based mental health services and hospitals were the care settings where Canadians and healthy systems benefited from renewing their prescription medication on the Web.
Resource saved (patient)	Canadians benefited by avoiding visits to primary and mental health care providers.	Canadians benefited by avoiding visits to primary and mental health care providers.	Canadians benefited by avoiding visits to primary health care providers.	Canadians benefited by avoiding visits to primary and mental health care providers.
Resource saved (health system)	Health systems benefited by increased productivity (time saved because of avoided visits and calls).	Health systems benefited by increased productivity (resources saved because of avoided emergency department visits).	Health systems benefited by increased productivity (resources saved because of avoided emergency department visits). Potential benefits were also identified through increased access via remote care provision.	Health systems benefited by increased productivity (time saved because of avoided visits) and increased quality (preventable adverse drug events avoided).

### Patient/Caregiver Perspective

#### Current Benefit

For patients and caregivers, direct value from PHRs currently manifests in the form of avoided in-person visits to health care providers, leading to savings in travel time and costs, caregiving costs and time arranging care, and time off work. These savings are generally shared across e-View, e-Visit, e-Rx renew, and virtual visit functionalities, with variations resulting from differing adoption rates, care settings, and the number of Canadians accessing certain types of health care services (ie, Canadians with a primary care physician or Canadians having a laboratory test in the past year).

**Figure 3:**
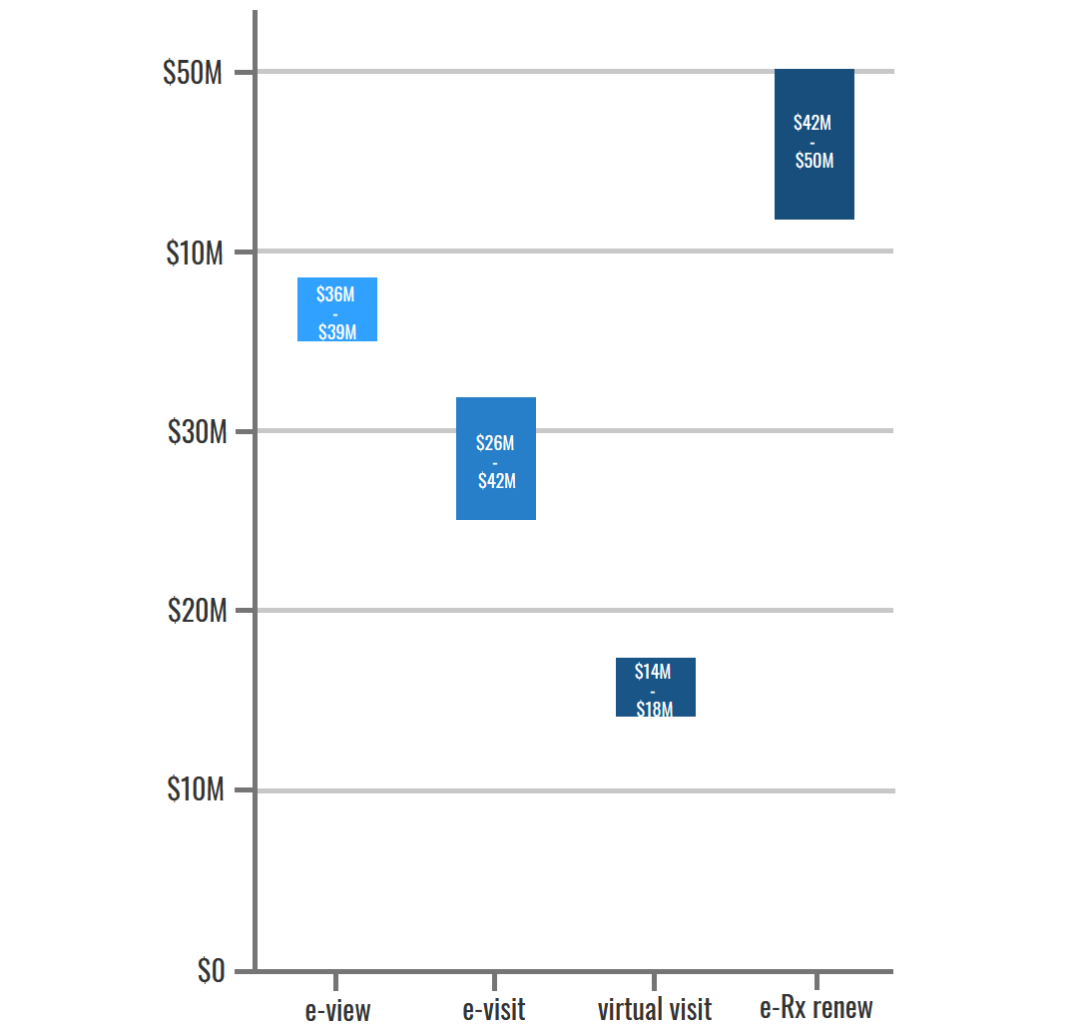
Current annual value to Canadians who use personal health records, by functionality. e-View: electronic view; e-Visit: electronic visit; e-Rx renew: electronic prescription renew.

Evidence of the value of PHRs for patients and caregivers in Canada has so far been demonstrated in primary care and mental health care settings (see Multimedia Appendix 9). Given this, model estimates were associated with population-level access estimates in these care settings. The value to Canadians who use currently available PHR functionalities is estimated to be Can $119 to Can $150 million per year (aggregated across all Canadians at current adoption rates). Figure 3 demonstrates the way in which this value is distributed across PHR functionalities: overall, our patient model demonstrates that most of the current value (2016-2017) to patients and caregivers is derived from e-Rx-renew (34%), followed by e-Visit (28%), e-View (26%), and virtual visit (12%).

Canadians have varying health care needs and, accordingly, different patterns of service use within the health care system. These factors affect the range and magnitude of value to citizens from use of the PHR functionalities estimated in this study and are important to interpret in context. We present below the range of total value for Canadian population subgroups based on frequency of visits per year [8], represented in terms of avoided costs because of avoided unnecessary in-person visits, weighted by utilization rates of PHR functionalities and the proportion of the population that reported each frequency of visit to their health care provider. The current annual aggregated value to Canadian patients/caregivers based on health system utilization for low-, medium-, and high-volume health system user segments of the Canadian population is as follows:

1. Can $61 to 153 million for the 41% of the Canadian population that makes 1 to 2 visits to a health care provider per year,
2. Can $87 to 148 million for the 19% of the Canadian population that makes 3 to 4 visits to a health care provider per year, and
3. Can $143 to 180 million or more for the 19% of the Canadian population that makes 5 or more visits to a health care provider per year (value estimates are based on 5 visits per year).

#### Potential Benefit

**Figure 4:**
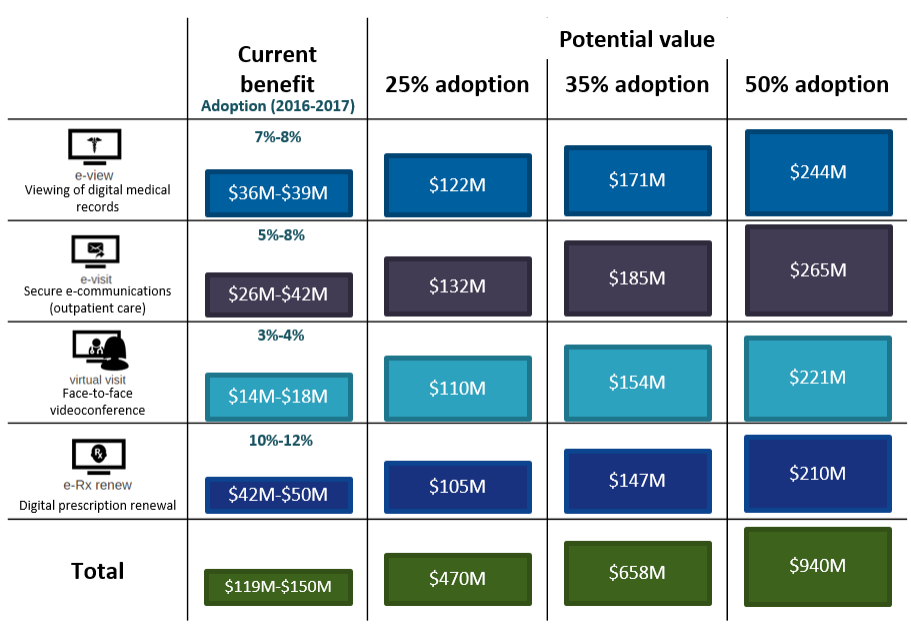
Projection of patient/caregiver benefits with increased personal health record adoption. e-view: electronic view; e-visit: electronic visit; e-Rx renew: electronic prescription renew.

On the basis of these value estimates, if Canadians were to increase PHR adoption from current adoption rates to 25%, 35%, or 50%, the total value to patients and caregivers would increase to Can $470 million, Can $658 million, and Can $940 million, respectively. Figure 4 represents how increasing adoption rates of each PHR functionality would increase the value from the patient perspective, assuming a steady-state of benefits realized (ie, that the benefits to Canadians remained constant over time).

### Health System Perspective

#### Current Benefit

From the health system perspective, we found that direct value from PHRs currently manifests in the form of increased productivity, leading to clinic or clinician time saved because of avoided visits and operational and clinician processes involved in responding to and resolving patient phone calls. There is also some evidence of increased health care quality, manifested by avoided preventable adverse drug events. Evidence of the value of PHRs for health systems in Canada has so far been demonstrated in primary care, hospital, pediatric, and mental health care settings, with value also identified internationally in other areas of specialized care (eg, veteran health services and chronic illnesses; see Multimedia Appendix 10). The non-Canadian evidence mentioned above also shows promising evidence of benefits for virtual visit functionalities.

**Figure 5:**
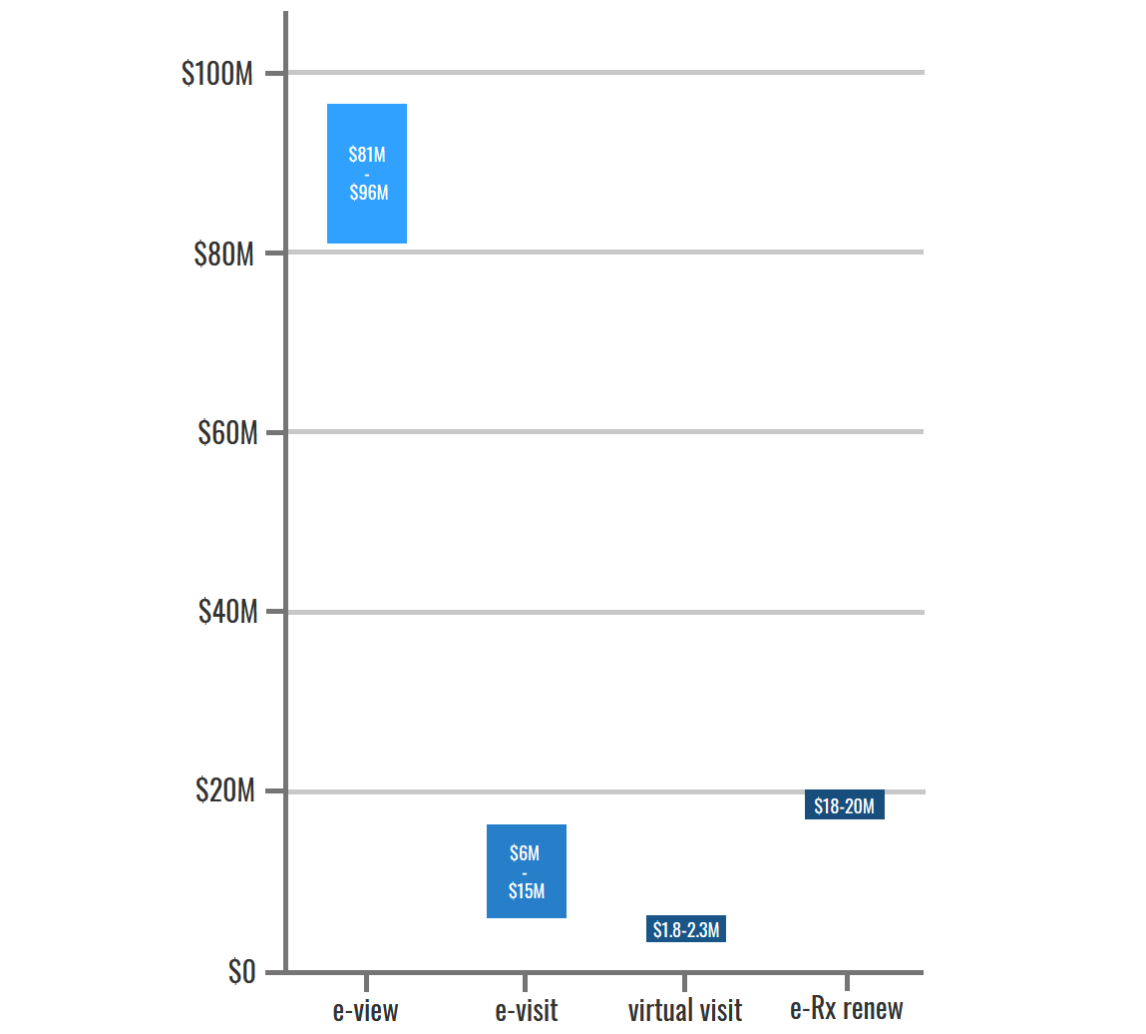
Current annual value to health systems where personal health record functionalities are in use. e-view: electronic view; e-visit: electronic visit; e-Rx renew: electronic prescription renew.

The current estimated value to health systems where PHR functionalities are in use is estimated to be Can $106 to Can $134 million a year. Figure 5 demonstrates the way in which that value is distributed across PHR functionalities. Overall, the health system model demonstrates value primarily related to e-View (72%)—the most widely available and utilized functionality—followed by e-Rx renew (15.2%), e-Visit (11.1%), and virtual visit (1.7%). We see below that most of the cost savings to the health system are driven by e-View functionality and are estimated at between Can $81 and Can $96 million per year.

Under the assumption that the savings associated with virtual visit use for specialized care can be realized in Canada at the same rate it has been realized internationally, savings for that functionality would rise from Can $27 to Can $54 million. In addition, in 2 of the identified studies, outcomes of PHR use were measured separately for different functionalities, allowing the model to explore the marginal benefit of increased PHR-enabled provider interaction within the same patient population. Using these studies, the model for valuing health system benefits of PHR use found that with increased opportunities for patients to directly consult or communicate with their providers, the value to the health system increased by 24% to 32%. In other words, moving from being able to passively view health care information on the Web to communicating or consulting virtually with a health care provider generated substantially more value to both patients and the health system. For example, in 1 study [31], the estimated benefit of patients using only e-Rx renew in a hospital setting was Can $105 million, whereas the estimated benefit of patients who used the e-Visit function was Can $127 million.

#### Potential Benefit

**Figure 6:**
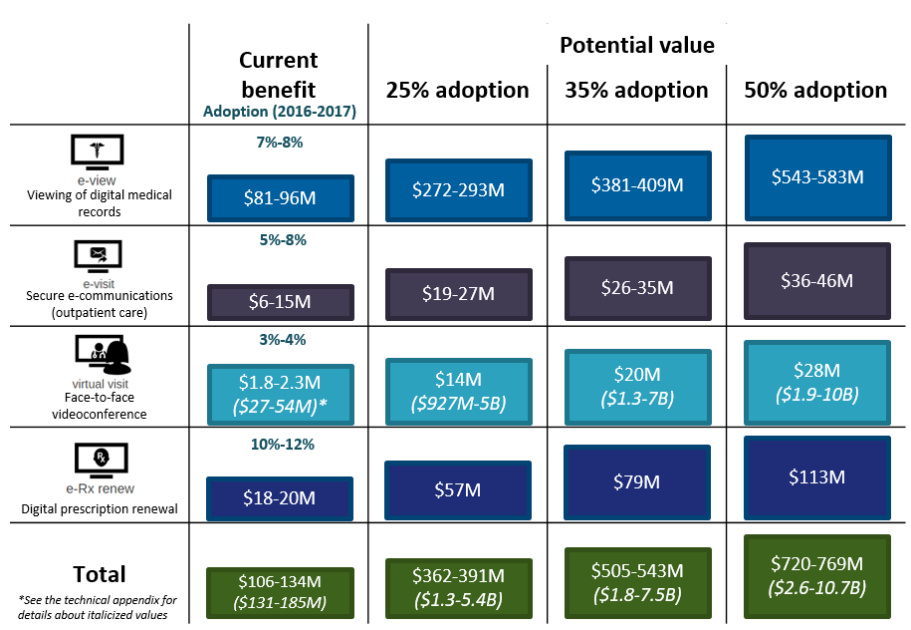
Projection of health system benefits with increased personal health record adoption. e-view: electronic view; e-visit: electronic visit; e-Rx renew: electronic prescription renew.

Using the estimates of value to health systems in Canada, we found that if Canadians were to increase PHR adoption to 25%, 35%, or 50%, the total value to patients and caregivers would increase to Can $362 to Can $391 million, Can $505 to Can $543 million, and Can $720 to Can $769 million, respectively. Figure 6 represents how increasing adoption rates of each PHR functionality would increase the value from the health system perspective, assuming a steady state of benefits realized. The second set of totals (represented by italicized figure ranges in parentheses below) represent the integration of benefits estimates that assume increased adoption and integrate evidence from jurisdictions outside of Canada with wrap-around PHR functionalities (inclusive of virtual visits). If Canadians had access to comparable PHRs, the total value to health systems in Canada would be Can $131 to Can $185 million at current adoption rates, and when projected to 25%, 35%, and 50%, the estimated values would range from Can $1.3 to Can $5.4 billion, Can $1.8 to Can $7.5 billion, and Can $2.6 to Can $10.7 billion, respectively.

### Societal Perspective

Most evidence regarding PHR use and health outcomes was generated in hospital or integrated care settings in the United States; 1 study was based in Canada. All of the evidence used as inputs into our population health model related to outcomes of interventions targeted specifically to people with diabetes or people with severe and persistent mental illness. Specific outcomes with sufficient evidence included improved health status through greater glycemic control, increased household income through improved life satisfaction, and increased positive health behaviors through improved medication adherence [21,26,40].

**Figure 7:**
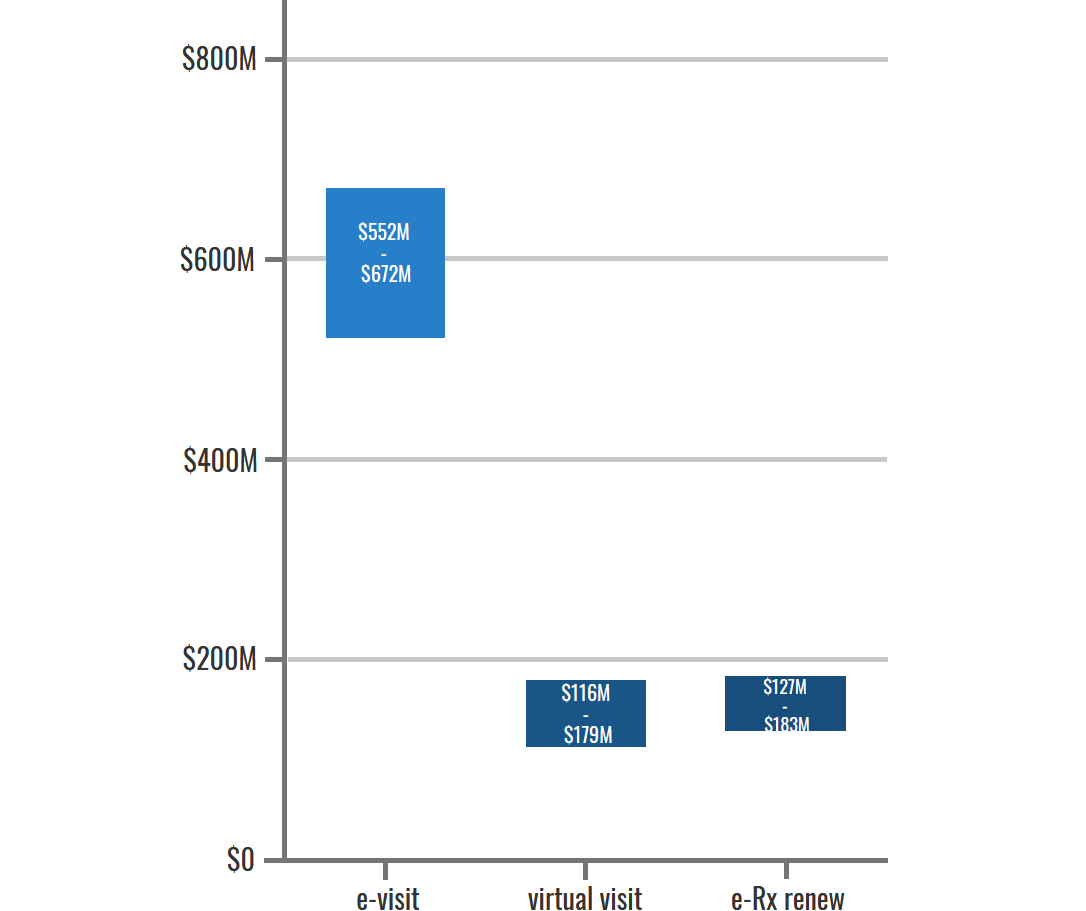
Value of improved health outcomes due to personal health record use, by functionality. e-view: electronic view; e-visit: electronic visit; e-Rx renew: electronic prescription renew.

The monetary value of those improved health outcomes breaks down as follows in Figure 7, with the majority of the economic benefit realized being driven by the e-Visit functionality.

Multimedia Appendices 9,10, and 11 provide summaries of each of the 3 quantitative models (patient/caregiver, health system, and population health).

## Discussion

Understanding how implementation of PHRs could benefit patients and caregivers, as well as health care systems in high-resource settings such as Canada, is an important step in exploring ways in which digital health technologies can facilitate improved patient engagement and, ultimately, population health outcomes and system transformation strategies for sustainability. We found that Canadians having Web access to their health care information, as well as communication and consultation services with providers via PHR functionalities, generates a substantial economic benefit from patient, health system, and broader societal perspectives. The hierarchy of classification factors we developed presents a practical approach to estimating economic benefit within the field of digital health and patient empowerment research, where questions of who accrues benefits and from which PHR functionalities are likely to inform decisions as to where and how increased access to PHR functionalities should be supported.

### Current Benefits

The majority of the current benefits seen in this study to Canadian patients and caregivers were realized in primary care and mental health settings and were resulting from the use of e-Rx renew functionalities *.* For health systems, the majority of the benefits were realized by citizens’ use of e-View functionalities within primary care settings. Comparing these 2 perspectives, the range of estimates for the health systems perspective was much wider than that of the patient/caregiver perspective (1.7%-72% vs 12%-34%, respectively). The relatively large share of benefits driven by e-Views for health systems, particularly in primary care settings, is likely a function of the limited available evidence, as well as the relatively broad population base that reports having access to primary care services in Canada. Patient access to health care information on the Web is therefore clearly linked to cost savings for both patients and health systems.

Evidence for benefits related to improved health outcomes was found in relation to PHR functionalities that enabled greater interaction and communication with health care providers and services: e-Visit, virtual visit, and e-Rx renew. This suggests that having access to technology that allows for more timely interactions between patients and health care providers and clinic administration has added benefits and value for the health of participating patients.

### Potential Benefits

For patient and health system perspectives, potential benefits were estimated using 2 different methods. The first method projected increases in adoption rates, and the second method relied on evidence from health management organizations in the United States that had implemented PHRs with integrated communication and clinical consultation e-service functionalities (e-Visit and virtual visits) [42,43]. Although such integrated PHR models are not yet available in Canada, the health outcomes reported resulted from patient use of PHR consultation and communication functionalities and may indeed be applicable to health outcomes in the Canadian context and that of other international health systems. Evidence from current and emerging models of care provides insight into key factors that facilitate health systems modernization to advance clinic/clinician adoption and integration of virtual care electronic services (e-Services), such as remuneration of health care providers.

The majority of potential improvement in health outcomes in our model is driven by increased integration of e-Services within health care organizations and by increased adoption of PHRs by older adults, particularly those in long-term care facilities. We were able to establish a direct link between increased life satisfaction for adults with severe and persistent mental illness who used PHRs compared with those who did not, with an equivalent increase in household income. This contributes to the literature by creating an empirical association between PHR use, patient activation and engagement [18], well-being, and ultimately economic benefit to populations accessing health care services.

### Process Matters—Effectiveness and Value of Personal Health Records in Context

Our findings are in line with other studies measuring the value of PHR use compared with business as usual or the absence of a PHR initiative within a health care organization [22,23,44]. There is added value of PHR implementation and adoption by patients and health care organizations, compared with settings with a lack of PHR access, but the degree to which realization of economic value offsets costs of investment and implementation of PHRs is difficult to assess. Similarly, it is challenging to understand the opportunity cost of investing in PHR implementation when benefit estimation perspectives and those sectors investing resources may not be wholly connected.

### Critical Mechanisms and Factors of Success

Ultimately, it is likely that PHRs with integrated communication and consultation functionalities will become business as usual in many health care settings and the suite of e-Services available will broaden to include a variety of automated machine learning and artificial intelligence features to support and advance health and wellness needs and facilitated interactions with care providers, organizations, and resources. As availability and adoption become more widespread, factors informing successful implementation, such as integration within health system workflows and provider remuneration structures, will become increasingly important.

### Limitations

We drew on evidence synthesized from the peer-reviewed literature as well as benefits evaluations of PHR implementation sites in Canada to develop 3 models estimating benefits of PHR use to Canadians and Canadian health systems. As such, our models are limited by the quality of the evidence generated from these studies. Variability in the number of respondents comprising study sample sizes, as well as the typical absence of a comparison group or counterfactual, also limits the generalizability of our estimates and may result in the over- or underestimation of benefits across the 3 models.

To estimate the value of PHR use from the patient/caregiver perspectives, we valued indirect costs using median income estimates in Canada and 50% of median hourly income to calculate nonlabor costs. It is likely that the distribution of income varies greatly across participants of the studies from which model inputs were drawn. As those who use PHRs tend to have higher income, this likely underestimates the value to current PHR users; however, it may more realistically represent the broader population who could potentially adopt PHRs.

To obtain a Canada-wide set of estimates, we used average costs for health services in Canada; however, these were weighted by the total Canadian population and appropriate adoption rates.

Finally, we did not include the cost of investment in PHR development, implementation, and evaluation, therefore, we do not make any claims about the relative benefit with respect to overall cost. We do, wherever possible, account for variable costs by using only the marginal benefit experienced by health care organizations to calculate estimates of economic value by perspective.

### Implications for Research and Practice

PHRs with high levels of direct interaction with care teams showed promising potential value if broadly implemented in the Canadian context with a particular focus on advancing adoption among certain clinical populations—namely, high-frequency health system users and their caregivers. Gaps in evidence include information about PHR use and related outcomes across PHR functions, care settings, and patient populations (see Table 3). From the patient/caregiver perspective, the care settings of studies that generated value-based estimates across e-Services did not include in-patient hospital care. Further research is needed to understand the value to patients and caregivers of PHR use when interacting with acute in-patient care settings. From the health system perspective, gaps in evidence available for estimating values differed according to PHR functionality. To gain a more fulsome understanding of how viewing medical records benefits both patients and health systems, research is needed to evaluate how this may differ across primary care versus specialized in-patient and outpatient hospital settings. There is also a paucity of information available concerning e-Visit, virtual visit, and e-Rx renew service use and related benefits. Finally, an important area for further research is understanding if and how PHR functionalities—particularly virtual visit—influence health system utilization and health outcomes for individuals with chronic conditions, especially older cohorts of adults.

**Table 3:** Current gaps in evidence related to personal health records.

Personal health record functionality	Electronic visit	Virtual visit	Electronic prescription renew
Care settings with evidence included	Community-based mental health services	Primary care	Community-based mental health and outpatient hospital services
Lack of evidence to inform model	Primary and outpatient specialist care	Outpatient specialist care	Primary care
Priority research area	Yes	Yes	Yes

Overall, more evidence is needed to expand the scope of benefits to include care settings outside of primary care and specialized mental health services, as well as settings advancing access to connected care e-service functionalities (beyond e-View). Understanding the different ways in which different populations benefit—and in which care settings—from saving resources because of PHR use will require further data development, especially regarding:

1. How caregivers use PHRs and how they benefit,
2. How geographic location and proximity to health services influences value for patients,
3. How key determinants of technology adoption may influence who and how patients, caregivers, and health systems use PHRs, and how this relates to any outcomes experienced, and
4. How different populations with high potential value approach PHR use and technology use more broadly.

### Conclusions

There is clear value to patients, health systems, and society of patients having Web-based access to their health care information and to consultation and communication e-Services with their providers and care organizations. Increasing this value and benefit would include bridging the digital divide and helping to facilitate training for both patients/caregivers and providers/organizations. To our knowledge, this is the first study synthesizing and estimating the value-based outcomes of PHR adoption and use across multiple sites at a national level. In addition, we feel we have contributed to the field by generating care setting and patient population–specific estimates across a range of adoption rates, effectiveness, and cost levels to explore how targeting various populations (eg, high-volume users, adults with severe and persistent mental illness, older adults in long-term care facilities, and individuals with chronic conditions) could yield further economic and health benefits to patients, caregivers, and health systems. Finally, our findings point to priority areas for new research that can allow for robust economic evaluations of PHRs, guide strategic health system policy and strategy, and identify empirical links between PHR use and cost savings, as well as determinants and mediators of PHR use and individual/population health outcomes across modernizing health care systems around the world.

## Appendix

Multimedia Appendix 1 Source list, patient/caregiver perspective.

Multimedia Appendix 2 Source list, health system perspective.

Multimedia Appendix 3 Studies and outcomes included for patient/caregiver perspective.

Multimedia Appendix 4 Studies and outcomes included in health system perspective.

Multimedia Appendix 5 Studies and outcomes included in population health perspective.

Multimedia Appendix 6 Narrative summary of patient/caregiver perspective estimates.

Multimedia Appendix 7 Narrative summary of health system perspective estimates.

Multimedia Appendix 8 Narrative summary of population health perspective estimates.

Multimedia Appendix 9 Quantitative model summary, patient/caregiver.

Multimedia Appendix 10 Quantitative model summary, health system.

Multimedia Appendix 11 Quantitative model summary, population health.

## Abbreviations

EHR: electronic health record
e-Rx renew: electronic prescription renew
e-Services: electronic services
e-View: electronic view
e-Visit: electronic visit
PHR: personal health record
