# Supplementary material for: Valuing Citizen Access to Digital Health Services: Applied Value-Based Outcomes in the Canadian Context and Tools for Modernizing Health Systems
Source: J Med Internet Res. 2019 Jun 6;21(6):e12277. doi: 10.2196/12277 (PMC6592482; doi:10.2196/12277)
Supplement: Multimedia Appendix 3 [file jmir_v21i6e12277_app3.docx]

Appendix 3: Studies and outcomes included for patient/caregiver perspective

| Studies included  (Patient/caregiver perspective) | Jurisdiction | PHR functions | Tangible outcome metrics | | |
| --- | --- | --- | --- | --- | --- |
|  |  |  | Revenues | Labour savings | Resource utilization |
| Canada Health Infoway. (2016). 2016 General public survey of Canadians: CHS short form. | Canada | e-view |  |  | Proportion of study users who accessed their medical records electronically in the past year |
|  |  | e-visit | Proportions of those who avoided an in-person visit who:   - Saved travel costs, and the mean costs saved - Saved caregiving costs and the mean costs saved |  | Proportion of uses who consulted or communicated with a medical professional online in the past year, and of those, the proportion who avoided an in-person visit as a result.  Of those that avoided an in-person visit, the proportion who:   - Saved travel time, and the mean time and cost saved |
|  |  | Virtual visit | Proportion of those who avoided an in-person visit who saved caregiving costs as a result and the mean costs saved | Proportion of those who avoided taking time off work as a result of an in-person visit and the mean time saved | Proportion of users who visited virtually with a medical professional in the past year, and who avoided an in-person visit as a result.  Proportion of those whose avoidance of an in-person visit led to saved travel time, and the mean value saved |
|  |  | E-Rx Renew |  |  | Proportion of users who had sent an electronic request for an Rx renewal in the past year, and who had requested that their doctor send an electronic request |
| Canada Health Infoway. (2017). ‘What Canadians Think’: Canadians’ perspectives on privacy of personal health information in the context of digital health. Final report. | Canada | e-view |  |  | Proportion of users who had accessed their medical records online, had viewed an online prescription list, and had had a lab test in the past year |
|  |  | e-visit |  |  | Proportion of users who had consulted with a medical professional and visited their doctor or place of care in the past year |
|  |  | Virtual visit |  |  | Proportion of users who had visited virtually with a medical professional, and visited their regular doctor or place of care in the past year |
|  |  | E-Rx Renew |  |  | Proportion of users who had requested that their doctor send a prescription renewal electronically, or who had used an online tool to request a prescription renewal in the past year |
| HQIC. (2016). miDASH, Consumer Health Solution. | Ontario, Canada | e-view | Proportion of those who avoided an in-person visit who:   - Saved travel costs and the mean cost saved - Saved caregiving costs and the mean cost saved | Proportion of those who avoided taking time of work as a result of an avoided in-person visit and the mean time saved | Proportion of users who avoided an in-person visit as a result of access to online medical records, and of those the proportion who:   - Saved travel time and mean time saved - Saved time they would have spent arranging caregiving and the mean time saved |
|  |  | E-Rx Renew | Proportion of those who avoided an in-person visit who:   - Saved travel costs and mean costs saved - Saved caregiving costs and mean costs saved | Proportion of those who avoided taking time of work as a result of an avoided in-person visit and the mean time saved | Proportion of those who avoided an in-person visit as a result of E-Rx Renew services, and of those the proportion who:   - Saved travel time and mean time saved - Saved time arranging caregiving and mean time saved |
| Warnar, K. & McConnachie, S. (2016). My health linked: Project review and benefits evaluation final report. | Ontario, Canada |  | Proportion of those who avoided an in-person visit who:   - Saved travel costs as a result of an avoided visit - Saved caregiving costs as a result of an avoided visit |  | Proportion of users who avoided an in-person visit because of e-view services, and of those the proportion who saved travel time as a result |
| Ontario Shores. (2016). Ontario Shores’ HealthCheck Patient Portal: Ontario Shores Centre for Mental Health Sciences Benefits Evaluation Report. | Ontario, Canada | e-view | Mean travel cost saved by avoided visits as a result of e-view services |  | Mean travel time saved by avoided visits as a result of e-view services |
|  |  | E-Rx Renew | Mean travel cost saved as a result of avoided visits |  | Mean travel time saved as a result of avoided visits |
