# Supplementary material for: Valuing Citizen Access to Digital Health Services: Applied Value-Based Outcomes in the Canadian Context and Tools for Modernizing Health Systems
Source: J Med Internet Res. 2019 Jun 6;21(6):e12277. doi: 10.2196/12277 (PMC6592482; doi:10.2196/12277)
Supplement: Multimedia Appendix 4 [file jmir_v21i6e12277_app4.docx]

Appendix 4: Studies and outcomes included in health system perspective

| Studies included  (health system) | Jurisdiction | PHR Functions | Tangible outcome metrics | | |
| --- | --- | --- | --- | --- | --- |
|  |  |  | Labour savings | Resource utilization | Healthcare service provision savings |
| Warnar, K. & McConnachie, S. (2016). My health linked: Project review and benefits evaluation final report. | Ontario, Canada | e-view | Avoided phone calls to primary care providers as a result of primary care PHR functions |  |  |
| Maxwell, J. (2016). Connect2care benefits evaluation – Results and final report. | Ontario, Canada | e-view | Avoided visits to primary care providers as a result of children’s hospital PHR functions |  |  |
| Epic EHR Program. (2015). Epic EHR Program: MyChart Consumer Health Solutions Benefits Evaluation Report (Pilot). | Ontario, Canada | e-view | Time saved by healthcare service providers in a children’s hospital |  |  |
| Social Research and Demonstration Corporation. (2015). Impacts of direct patient access to laboratory results – Final Report. Ottawa, ON: SRDC. | British Columbia, Canada | e-view | Avoided visits to primary care provider as a result of e-view services in primary care settings |  |  |
| Ontario Shores. (2016). Ontario Shores’ HealthCheck Patient Portal: Ontario Shores Centre for Mental Health Sciences Benefits Evaluation Report. | Ontario, Canada | e-view | Time saved through avoided requests for information in mental health settings |  |  |
|  |  | e-Rx Renew | Avoided visits to mental health providers |  |  |
| eHealth Saskatchewan. (2017). Citizen Health Information Portal (CHIP) Pilot Project: Benefits Evaluation Report. | Saskatchewan, Canada | e-view | Avoided visits to primary care providers in primary care settings |  |  |
| Slawomirski, L. A., Auraaen, A., & Klazinga, N. S. (2017). The economics of patient safety: Strengthening a value-based approach to reducing patient harm at national level. OECD Health Working Papers No. 96. Paris: OECD. | Canada and international | e-Rx Renew | Reduced preventable adverse drug events | Reduction in aggregate patient cost | Medical error avoided |
| Lawson Health Research Institute. (2014). Mental Health Engagement Network (MHEN): Connecting clients with their health team benefits evaluation report. | Ontario, Canada | e-visit | Avoided in-person visits to outpatient services in mental health care settings  Avoided visits to psychiatric emergency departments |  |  |
| Infoway. (2016). Virtual Visits in British Columbia: 2015 Patient Survey and Physician Interview Study. | British Columbia, Canada | Virtual visit | Avoided emergency department visits |  |  |
| Darkins, A., Ryan, P., Kobb, R., Foster, L., Edmonson, E., & Wakefield, B. (2008). Care Coordination/Home Telehealth: They systematic implementation of health informatics, home telehealth, and disease management to support the care of veteran patients with chronic conditions. Telemedicine and e-Health, 14(0), 1118-1126. | USA | Virtual visit | Reduction in numbers of bed-days of care | Reduction in cost per patient per annum of CHS vs. in-home PC  Reduction in cost per patient per annum of CHS vs. direct cost of institutional LTC |  |
| Sorondo, B., Allen, A., Fathima, S., Bayleran, J., & Sabbath, I. (2017). Patient portal as a tool for enhancing patient experience and improving quality of care in primary care practices. eGEMS (Generating Evidence & Methods to improve patient outcomes), 4(1). | USA | Virtual visit | Reduction in emergency department visits  Reduction in hospital admissions |  |  |
