# Supplementary material for: Valuing Citizen Access to Digital Health Services: Applied Value-Based Outcomes in the Canadian Context and Tools for Modernizing Health Systems
Source: J Med Internet Res. 2019 Jun 6;21(6):e12277. doi: 10.2196/12277 (PMC6592482; doi:10.2196/12277)
Supplement: Multimedia Appendix 5 [file jmir_v21i6e12277_app5.docx]

Appendix 5. Studies and outcomes included in population health perspective

| Studies included  (Population health-health system) | Jurisdiction | PHR functions | Intangible to tangible | | | |
| --- | --- | --- | --- | --- | --- | --- |
|  |  |  | Improved health outcome | Improved health behaviour outcome | Improved wellbeing | Value perspective |
| Shimada, S. L., Allison, J. J., Rosen, A. K., Fen, H., & Houston, T. K. (2016). Sustained Use of Patient Portal Features and Improvements in Diabetes Physiological Measures. J Med Internet Res, 18(7): e179. | USA | e-visit  e-Rx Renew | Improved glycemic control |  |  | Health systems  Patients/ caregivers |
| Lawson Health Research Institute. (2014). Mental Health Engagement Network (MHEN): Connecting clients with their health team benefits evaluation report. | Ontario, Canada | e-visit |  |  | Improved life satisfaction | Patients/ caregivers  Societal |
| Toscos, T., Daley, C., Heral, L., Doshi, R., Chen, Y-C., Eckert, G. J., Plant, R. L., & Mirro, M. J. (2016). Impact of electronic personal health record use on engagement and intermediate health outcomes among cardiac patients: a quasi-experimental study. J Am Med Inform Assoc, 23:119-128. | USA | e-visits | Improved glycemic control |  |  | Health systems  Patients/ caregivers |
| Shaw, R. J., & Ferranti, J. (2011). Patient-provider internet portals – Patient outcomes and use. Computers, Informatics, and Nursing, 29(12), 714-718. | USA | e-visits | Improved glycemic control |  |  | Health systems  Patients/ caregivers |
| Sakar , U., Lyles, C. R., Parker, M. M., Allen, J., Nguyen, R., Moffet, H.H., & Karter, A. J. Use of the refill function through an online patient portal is associated with improved adherence to statins in an integrated health system. Med Care, 52(3), 194-201. | USA | e-Rx |  | Decrease in medication non-adherence |  | Health systems  Patients/ caregivers |
