# Supplementary material for: Valuing Citizen Access to Digital Health Services: Applied Value-Based Outcomes in the Canadian Context and Tools for Modernizing Health Systems
Source: J Med Internet Res. 2019 Jun 6;21(6):e12277. doi: 10.2196/12277 (PMC6592482; doi:10.2196/12277)
Supplement: Multimedia Appendix 6 [file jmir_v21i6e12277_app6.docx]

**Appendix 6**

**Narrative summary of patient/caregiver perspective estimates**

The patient estimates were calculated to reflect a broad range of solution uses, as well as variability in the size of the potential population. Different estimates also reflected a variety of source materials, and their different methods of using the technology.

Explanation of the specific multipliers used for e-visit, e-view, and e-Rx renew estimates is expanded more fully below. However, all estimates were also based on a number of more generalized multipliers. Data for the proportion of users who avoided a visit due to solution use, and who saved costs as a result of avoiding a visit, were based on Canada Health Infoway (CHI) and Health Quality Innovation Collaborative (HQIC) data. When estimating costs saved as a result of saved time (such as saved travel time), two options were presented based on the assumption that personal time can be valued at between 25 per cent and 50 per cent of an individual’s salary, based on a median of Canadian salaries identified by Statistics Canada. Population data was also provided by Statistics Canada, and applied to multipliers based on the population of care:

- For full population estimates, the full population of the jurisdiction was used;
- For primary care estimates, the population of the jurisdiction was applied to a multiplier representing the percentage of Canadians with a regular source of care. Two options were provided, an estimate of 82% from the Health Council of Canada, and an estimate of 91% from CIHI;
- For mental health care, the population used was the population with mental or substance disorders, by jurisdiction, as reported by Statistics Canada.

E-view estimates included usage estimates for accessing records electronically, from 2016 Canada Health Infoway data, and viewing vaccine/immunization lists online, accessing medical records online, viewing online prescription lists, and viewing online lab tests, all from 2017 Canada Health Infoway data. In determining the potential value of the solution to the full population, a multiplier was also applied to estimates based on the proportion of people who reported having a lab test in the past year, from Canada Health Infoway 2017 data. The mean cost saved used both data from HQIC (for estimates based on a primary care function), and from Ontario Shores (for estimates based on mental health service use).

Estimates at the higher end with respect to value represent the value of viewing online lab tests, while estimates at the low end with respect to value represent the value of viewing online vaccine/immunization lists, both based on 2017 Canada Health Infoway data. For example, the high-end estimate for saved travel costs for patients using e-view solutions in Ontario for primary care services was calculated as follows:

Savings Estimate: Saved travel costs for Ontario patients using e-view solutions in Ontario for primary care services

e-Rx renew renew estimates included usage estimates for sending a request electronically for a prescription renewal and requesting that your doctor send a prescription renewal electronically, both from Canada Health Infoway 2016 data, and requesting that your doctor send a prescription electronically and using an online tool to request a prescription renewal, both from Canada Health Infoway 2017 data. In determining the potential value of the solution to the full population, a multiplier was also applied to estimates based on the proportion of people who reported filling a prescription in the past year, from Canada Health Infoway 2017 data. The mean cost saved used both data from HQIC (for estimates based on a primary care function), and from Ontario Shores (for estimates based on mental health service use).

Estimates at the high end with respect to value represent the value of requesting that your doctor send a prescription electronically, based on 2017 Canada Health Infoway data, while estimates at the low end with respect to value represent the value of sending a request electronically for a prescription renewal, based on 2016 Canada Health Infoway data. For example, the high-end estimate for saved caregiving costs for patients using e-Rx renew solutions in Ontario for primary care services was calculated as follows:

Savings Estimate: Saved caregiving costs for Ontario patients using e-Rx renew solutions in Ontario for primary care services

E-visit estimates included usage estimates for both consulting/communicating with service providers online, based on Canada Health Infoway data from both 2016 and 2017. In determining the potential value of the solution to the full population, a multiplier was also applied to estimates based on the proportion of people who reported visiting their regular doctor or place of care in person in the past year, from Canada Health Infoway 2017 data. The mean cost saved used both data from Canada Health Infoway (for estimates based on the full population), and from Ontario Shores (for estimates based on mental health service use).

Estimates at the higher end with respect to value represent the value of consulting or communicating online, based on 2016 Canada Health Infoway data, while estimates at the low end with respect to value represent the value of based on 2017 Canada Health Infoway data. For example, the high-end estimate for saved travel time for patients using e-visit solutions in Ontario for mental health services was calculated as follows:

Savings Estimate: Saved travel time for patients in Ontario using e-visit solutions in Ontario for mental health services

The model includes a highlighted an estimate for each solution, organization, jurisdiction and resource saved that represents the estimate that has the most alignment and consistency across variables, as follows:

- For e-views, the viewed online lab test estimates for solution use (CHI, 2016) and the HQIC estimates for saved costs, where available;
- For e-Rx renew, the estimates for using an online tool to request a prescription renewal (CHI, 2017), and the HQIC and Ontario Shores (mental health) estimates for saved costs;
- For e-visits, the consult/communicate online estimates (CHI, 2016);
- An estimate for saved personal time of 50% of the median Canadian income;
- An estimate that 91% of the Canadian population say they have a regular source of care.
