# Supplementary material for: Valuing Citizen Access to Digital Health Services: Applied Value-Based Outcomes in the Canadian Context and Tools for Modernizing Health Systems
Source: J Med Internet Res. 2019 Jun 6;21(6):e12277. doi: 10.2196/12277 (PMC6592482; doi:10.2196/12277)
Supplement: Multimedia Appendix 7 [file jmir_v21i6e12277_app7.docx]

**Appendix 7**

**Narrative summary of health system perspective estimates**

The model estimating the benefits for health system payers across Canada accounted for healthcare and population-related variables, and weighted estimates of benefit value by the proportion of the population for whom each mode of action was plausibly applicable. SRDC’s model used the domains outlined in the Benefits Estimation framework provided by Infoway – healthcare Quality, Access, and Provider/System productivity to categorize outcomes from the payer perspective. These outcomes ranged from avoided costs and visits reported by those using PHRs in the Infoway and peer-reviewed literature/studies, to increases in health system capacity, and to ‘tree top’ outcomes including healthcare and health behavior and health status outcomes.

Variables included care setting where the PHR was introduced (e.g. primary care practice, tertiary hospital); population targeted in PHR initiatives (e.g. children with diabetes, adults with mental disorders); and other population parameters (jurisdiction-specific, adoption rates). Statistics Canada, Infoway, and other relevant sources were used to calculate population weight estimates; Canadian Institute for Healthcare Information (CIHI) and peer-reviewed literature were used to calculate the value of payer benefits derived from PHR initiatives.

For each estimate, we used information specific to the province in which the evidence was generated in order to value the benefit of PHRs. Certain outcomes included in the model were only available at the national-level, or were sourced from other countries, and adjusted accordingly. We will provide a step-by-step ‘walk through’ of the types of calculations we employed to generate the final estimates.

**Estimates generated by Infoway studies**

*Approach:*

- Infoway study 🡪 avoided service use 🡪 population specific services 🡪 proportion of province with relevant medical needs 🡪 cost*number of pop-specific services🡪outcome – proportion of avoided services
  - Assumes: Proportion of those who avoid a visit in the study population will avoid a visit in the larger relevant population of that province, conditional on the proportion of the population in that province with access to and reported use of the technology.
  - If the study population is comprised of adults with severe mental health disorders (ON Shores) we estimate the value of the benefit by examining what proportion of Ontarians have similar issues; what services they avoided in the study, and what the rate of these services are used for the relevant population, and then what the proportion of the population currently can and has accessed this type of digital health solution. Some estimates are calculated at the individual provider-level, some at the organizational-level and some at the health system level depending on the unit of analysis of the source study.

**E-view estimates**

PHR e-view functionalities generated the following benefits from the payer perspective:

*Because patients are able to access their healthcare information via their PHR, organizations benefit from:*

- Increased capacity due to time saved by administrative and healthcare staff
  - Evidence for increased capacity for healthcare organizations came from a children’s hospital (CHEO^1^), and a tertiary hospital specializing in mental healthcare (ON Shores^2^), both located in Ontario.

*Because patients are able to access their healthcare information via their PHR, healthcare providers benefit from:*

- Increased practice capacity due to avoided phone calls from patients
- Increased practice capacity due to avoided in-person visits from patients
  - Evidence for increased practice capacity resulting from avoided phone calls came from a children’s hospital in ON (CHEO^1^); and a Family Health Team group of primary care practices in ON (Barrie Group FHT^3^).
  - Evidence for increased practice capacity resulting from avoided in-person visits came from a children’s hospital specializing in injury and rehabilitation (Bloorview^4^); and two studies related to primary care settings (CHIP-SK^5^ and HealthLink BC^6^). The PHR initiatives were located in Ontario, Saskatchewan, and BC respectively.

**E-Rx Renew estimates**

PHR e-Rx renew functionalities generated the following benefits from the payer perspective:

*Because patients are able to view and renew their prescription information online, the healthcare system benefits from:*

- Increased healthcare quality resulting from reduced preventable adverse drug events (pADEs) or medication-related error in hospital settings
  - Evidence for this improved quality care came from an OECD study of high-income countries with Canada-specific evidence regarding PHRs’ effects on pADE reduction^7^.

*Because patients are able to view and renew their prescription information online, healthcare providers benefit from:*

- Increased practice capacity resulting from reduced in-person visits from patients
  - Evidence for this increase in system productivity came from a tertiary hospital specializing in mental healthcare (ON Shores^2^), located in Ontario.

**E-visit estimates:**

PHR e-visit functionalities generated the following benefits from the payer perspective:

*Because patients with severe and persistent mental health are able to contact their healthcare provider network, the healthcare system benefits from:*

- Increased practice capacity resulting from avoided in-person visits to hospital outpatient units
- Increased practice capacity resulting from avoided in-person visits to psychiatric emergency departments^[[1]](#footnote-1)^
  - Evidence for this increased practice capacity came from a study of a network of healthcare professionals providing mental health services to adults with severe and persistent mental disorders in Ontario (Mental Health Engagement Network^8^).

*Because patients with chronic conditions are able to access their healthcare providers/network of providers via their PHR, healthcare systems benefit from:*

- Patients’ increased access to healthcare providers virtually, as opposed to in-home, face-to-face support
- Patients’ increased access to healthcare providers virtually at home, as opposed to being transferred into long-term care facilities
- Patients’ increased access to upstream care by providers resulting in reduced bed-days of care in hospitals
- Patients’ increased access to upstream care by providers resulting in a reduction of emergency department visits
- Patients’ increased access to upstream care by providers resulting in a reduction of hospital admissions^[[2]](#footnote-2)^
  - Evidence for a reduction in costs resulting from virtual contact with providers came from the Veteran’s Affairs group in the United States^9^.
  - Evidence for reduced numbers of emergency department visits and hospital admissions came from the Kaiser Permanente group in the United States^10^.

For the most part, the estimates generated reflect current or actual value of a study, expanded to a plausible current or actual benefit at the provincial or national level, depending on the unit of analysis from the source evidence. We also present projections of potential benefit, should adoption rates increase over time, provincially and nationally.

Key assumptions made for actual/current estimates include:

- The benefits realized by the study sample are reflective of benefits populations with similar case-mixes or diagnoses would realize;
- Benefits realized by healthcare organizations in terms of increased productivity, are expandable to other, similar contexts across Canada;
- The proportion of the population that each province in Canada represents, can be used to expand province-level benefits to a national-level benefit, and in some cases, to disaggregate into provincial-level benefits.
- All estimates generated by valuing provider time, or time-saved to providers and healthcare organizations, were done so by creating a range of values from the least expensive unit of time (e.g. an administrative staff), to the most expensive unit of time (a physician).
- For projections, we assume a steady-state of PHR effect over time.

**References**

1. Children’s Hospital of Eastern Ontario. *Epic EHR Program: MyChart Consumer Health Solutions Benefits Evaluation Report (Pilot) CHS-09B / CHSONCHEO02*. Ottawa, ON; 2015. https://www.infoway-inforoute.ca/en/component/edocman/3091-epic-ehr-program-mychart-consumer-health-solutions-benefits-evaluation-report-pilot/view-document?Itemid=101.

2. Canada Health Infoway. *Ontario Shores ’ HealthCheck Patient Portal Ontario Shores Centre for Mental Health Sciences Benefits Evaluation Report Table of Contents*. Toronto, ON; 2016.

3. Warnar K, McConnachie Se. *Myhealthlinked: Project Review and Benefits Evaluation Final Report*. Barrie, ON; 2016.

4. Bloorview H, Report BE. connect2care Benefits Evaluation – Results and Final Report. 2016;(June):1-43.

5. eHS CHIP Benefits Evaluation Report 2017_V1.

6. Social Research and Demonstration Corporation. Impact of direct patient access to laboratory results. *Soc Res Demonstr Corp*. 2015:52. https://www.infoway-inforoute.ca/en/component/edocman/2775-impacts-of-direct-patient-access-to-laboratory-results-final-report/view-document?Itemid=101.

7. Slawomirski L, Auraaen A, Klazinga N. The Economics of Patient Safety. *Oecd*. 2017;(March):194-205. doi:doi:10.1201/9781420051254.ch5

8. Norman R, Mitchell B. *Mental Health Engagement Network (MHEN): Connecting Clients with Their Health Team*.; 2012.

9. Darkins A, Ryan P, Kobb R, Foster L, Edmonson E, Wakefield B. Care coordination/home telehealth: The systematic implementation of health informatics, home telehealth, and disease management to support the care of veteran patients with chronic conditions. *Telemed e-Health*. 2008;14(10):p.1118.

10. Sorondo B, Allen A, Fathima S, Bayleran J. Patient Portal as a Tool for Enhancing Patient Experience and Improving Quality of Care in Primary Care Practices. *eGEMs J Electron Heal Data Methods*. 2017;4(1).

1. We included benefits generated from avoided visits to hospitals and emergency departments, and not to healthcare professionals in the network, as it was unclear to what extent e-visits with the network professionals’ utilized physician or other professionals’ time. We therefore included visits to healthcare services that would not be provided by the network. [↑](#footnote-ref-1)
2. Because outcomes for these estimates were sourced from networks of care in the US, we adjusted the magnitude of these outcomes in two ways: first, we adjusted the dollar amount saved by the ratio of healthcare expenditure as a proportion of GDP per capita for the US vs. Canada; and second we assumed a 25% to 50% ‘cap’ on the magnitude of the outcome as the VA and KP portals are highly sophisticated with high adoption rates. We made these adjustments to account for health system and incentive differences for patients and providers. [↑](#footnote-ref-2)
