# Supplementary material for: Valuing Citizen Access to Digital Health Services: Applied Value-Based Outcomes in the Canadian Context and Tools for Modernizing Health Systems
Source: J Med Internet Res. 2019 Jun 6;21(6):e12277. doi: 10.2196/12277 (PMC6592482; doi:10.2196/12277)
Supplement: Multimedia Appendix 8 [file jmir_v21i6e12277_app8.docx]

**Appendix E**

**Narrative summary of population health perspective estimates**

**Conceptual framework**

PHRs can benefit patients and healthcare systems by improving health and healthcare-related behaviours. Risling et al. have conceptualized the mechanisms by which, and cyclical nature of patient empowerment-activation-engagement via digital health solutions^1^. Hibbard et al. have developed a framework hypothesizing the causal link between patient characteristics, activation, and subsequent health-related outcomes^2^. We combine these conceptual frameworks and using outcomes from Infoway PHR initiative evaluations, and other jurisdictions with PHR interventions, we estimate the value of the effects PHR use can have on population health.

Estimates of benefit to healthcare systems in Canada, where there are tangible costs avoided to the payer (avoided usage of provider time through communication and in-person visits) attributable to PHRs, are more straightforward than less tangible benefits such as improved health and healthcare behaviours and health status outcomes. In order to provide estimates of improvements to behaviours and health status, we employed techniques to convert benefits reported in the literature/evidence base we amassed, into monetary values. We outline our approaches to monetizing these benefits, as well as assumptions related to each method.

*E-Rx renew benefits*

The main source of benefits resulting from PHRs with e-Rx renew functionalities, was increased medication adherence, and improved glycemic control for those with diabetes.

*Because patients with diabetes can view and renew their prescriptions via their PHR, patients and, ultimately the healthcare system benefit from:*

- Increased quality healthcare, resulting in improved health behavior and health status outcomes
  - Evidence for improved medication adherence and glycemic control for people with diabetes came from a study based in an integrated care setting^3^, and a public hospital setting^4^ both based in the United States.
- Benefits were valued using estimates of the cost of medication non-adherence of an individual with diabetes, to healthcare systems^5,6^, weighted by adoption rates, and the proportion of the Canadian population with diabetes currently taking diabetes-related medication. Other inputs to valuing these estimates, was a study that calculated the monetary benefit (via avoided costs) of improved health behaviours of those with diabetes. Benefits to society were estimated in terms of patients’ ability to remain in the labour force as a result of maintaining glycemic control.
- The range reflects the high and low values estimated for costs and benefits to the healthcare system.
- One study found that e-Rx renew renewal functionality generated a benefit for PHR users, but that the added functionality of e-messaging resulted in even more health benefits for users^4^ (see below), allowing us to calculate the *marginal* benefit of increased engagement via PHRs.

*E-visit benefits: E-messaging*

E-messaging with providers was found to be more beneficial to PHR users than e-Rx renew. This suggests that having access to technology that allows for more engaged interactions between patients and healthcare providers has added benefits and value. We present the incremental and marginal benefits of increasing PHR functionality from e-Rx renew view and renew capabilities to e-messaging with providers for those with diabetes.

*Because patients with diabetes can message securely with their healthcare providers via their PHR, patients, and ultimately the healthcare system benefit from:*

- Increased quality healthcare, resulting in improved health behavior and health status outcomes
- Increased ability of patients to maintain their participation in the labour force
- In general PHRs that facilitated direct contact with providers as opposed to those with just e-view and e-Rx renew functionalities, benefited social sectors up to 2.5 times more (between 25% and 250%) ^4,7^.

*E-visit benefits: E-visits with providers*

In order to monetize the reported improvement of life satisfaction as a result of PHR use by adults with severe and persistent mental illness, we applied a compensating differential approach developed by Helliwell and Huang (2010)^8^. This method allows increases or decreases in the 10-item General Life Satisfaction scale to be converted into household income equivalents. In order to convert the PHR-related change in life satisfaction to household income, we take the difference in life satisfaction pre/post for those in the MHEN study (0.27) and then divide this by the coefficient for the marginal effect of life satisfaction on household income for Canadians (β=0.14). This provides value by which we multiply individuals’ household incomes by in order to estimate the dollar value of increased life satisfaction via PHR. We assume that individuals have a net income of $30,000^[[1]](#footnote-1)^, and then calculate the tax revenue generated by the equivalent increase in household income (20.05% of the total). We then multiply the tax revenue generated by, for the minimum estimated value, the number of individuals in the source study (MHEN n=394); and for the maximum estimated value, the number of individuals in the province of Ontario who report having a mood or organic mental disorder (n=377,039^[[2]](#footnote-2)^).

**References**

1. Risling T, Martinez J, Young J, Thorp-Froslie N. Evaluating Patient Empowerment in Association With eHealth Technology: Scoping Review. *J Med Internet Res*. 2017;19(9):e329. doi:10.2196/jmir.7809

2. Hibbard JH, Greene J, Becker ER, et al. Racial/ethnic disparities and consumer activation in health. *Health Aff*. 2008;27(5):1442-1453. doi:10.1377/hlthaff.27.5.1442

3. Sarkar U, Lyles CR, Parker MM, et al. Use of the refill function through an online patient portal is associated with improved adherence to statins in an integrated health system. *Med Care*. 2015;52(3):194-201. doi:10.1097/MLR.0000000000000069.Use

4. Lyles CR, Sarkar U, Schillinger D, et al. Refilling medications through an online patient portal : consistent improvements in adherence across racial / ethnic groups. 2016;(415):28-33. doi:10.1093/jamia/ocv126

5. Sokol MC, McGuigan KA, Verbrugge RR, Epstein RS. Impact of medication adherence on hospitalization risk and healthcare cost. *Med Care*. 2005;43(6):521-530.

6. Testa M, Simonson D. Health economic benefits and quality of life during improved glycemic control in patients with type 2 diabetes mellitus: A randomized, controlled, double-blind trial. *JAMA*. 1998;280(17):1490-1496. http://dx.doi.org/10.1001/jama.280.17.1490.

7. Bloorview H, Report BE. connect2care Benefits Evaluation – Results and Final Report. 2016;(June):1-43.

8. Helliwell JF, Huang H. How’s the Job? Well-Being and Social Capital in the Workplace. *Ind Labour Relations Rev*. 2010;63(10):205-227. doi:10.1177/001979391006300202

9. Poremski D, Distasio J, Hwang SW, Latimer E. Employment and income of people who experience mental illness and homelessness in a large canadian sample. *Can J Psychiatry*. 2015;60(9):379-385. doi:10.1177/070674371506000902

1. It’s likely that individuals with severe mental illness make less – one study estimates that 67% of individuals with mental illness source their income primarily from social assistance, the median monthly amount of which is $412 for individuals in Ontario^9^. This would mean a yearly income of just under $5000. Further investigation is needed to explore estimating the cost-savings from the government’s perspective of income primarily sourced from social assistance as these are often imputed and over/understated. [↑](#footnote-ref-1)
2. All population estimates come from CANSIM tables provided by Statistics Canada and are referenced in Appendix C. [↑](#footnote-ref-2)
